# Proton Magnetic Resonance Spectroscopic Evidence of Glial Effects of Cumulative Lead Exposure in the Adult Human Hippocampus

**DOI:** 10.1289/ehp.9645

**Published:** 2007-01-03

**Authors:** Marc G. Weisskopf, Howard Hu, David Sparrow, Robert E. Lenkinski, Robert O. Wright

**Affiliations:** 1 Department of Environmental Health, Harvard School of Public Health, Boston, Massachusetts, USA; 2 The Channing Laboratory, Department of Medicine, Brigham and Women’s Hospital, and Harvard Medical School, Boston, Massachusetts, USA; 3 VA Boston Healthcare System, Boston, Massachusetts, USA; 4 Boston University School of Medicine, Boston, Massachusetts, USA; 5 Department of Radiology, Beth Israel Deaconess Medical Center, Boston, Massachusetts, USA

**Keywords:** bone lead, choline, glia, hippocampus, myoinositol, *N*-acetylaspartate, neuronal viability, proton MRS

## Abstract

**Background:**

Exposure to lead is known to have adverse effects on cognition in several different populations. Little is known about the underlying structural and functional correlates of such exposure in humans.

**Objectives:**

We assessed the association between cumulative exposure to lead and levels of different brain metabolite ratios *in vivo* using magnetic resonance spectroscopy (MRS).

**Methods:**

We performed MRS on 15 men selected from the lowest quintile of patella bone lead within the Department of Veterans Affairs’ Normative Aging Study (NAS) and 16 from the highest to assess in the hippocampal levels of the metabolites *N*-acetylaspartate, myoinositol, and choline, each expressed as a ratio with creatine. Bone lead concentrations—indicators of cumulative lead exposure—were previously measured using K-X-ray fluorescence spectroscopy. MRS was performed on the men from 2002 to 2004.

**Results:**

A 20-μg/g bone and 15-μg/g bone higher patella and tibia bone lead concentration—the respective interquartile ranges within the whole NAS—were associated with a 0.04 [95% confidence interval (CI), 0.00–0.08; *p* = 0.04] and 0.04 (95% CI, 0.00–0.08; *p* = 0.07) higher myoinositol-to-creatine ratio in the hippocampus. After accounting for patella bone lead declines over time, analyses adjusted for age showed that the effect of a 20-μg/g bone higher patella bone lead level doubled (0.09; 95% CI, 0.01–0.17; *p* = 0.03).

**Conclusions:**

Cumulative lead exposure is associated with an increase in the myinositol-to-creatine ratio. These data suggest that, as assessed with MRS, glial effects may be more sensitive than neuronal effects as an indicator of cumulative exposure to lead in adults.

Adverse effects of elevated blood and bone lead levels on cognitive function have been examined in various populations including children (Banks et al. 1997; Canfield et al. 2003; Needleman and Gatsonis 1990; Schwartz 1994), occupationally exposed adults (Balbus-Kornfeld et al. 1995; Bleecker et al. 1997; Fiedler et al. 2003; Schwartz and Stewart 2000; Stewart et al. 1999), and nonoccupationally exposed adults (Muldoon et al. 1996; Payton et al. 1998; Weisskopf et al. 2004b; Wright et al. 2003). These effects have been assessed using primarily behavioral and neuropsychologic evaluations and are some of the most consistently reported impairments associated with lead exposure. Regarding the molecular and cellular effects of lead exposure that may underlie the behavioral effects, there is a good deal of data from experimental studies in animals, but relatively little is known about the structural and functional correlates of lead-related brain dysfunction in humans. A better understanding of the underlying pathology would not only have potential benefits for prevention and treatment of the adverse effects but could also help identify those effects specifically attributable to lead.

Magnetic resonance spectroscopy (MRS) provides a noninvasive method with which to monitor biochemical aspects of acute and chronic stages of neurologic disease in the human brain (Ross et al. 2006). The development of spatially localized spectroscopic methods that sample the relative levels of metabolites from volumes of tissue defined from magnetic resonance imaging (MRI) scans has provided a basis for integrating the biochemical information obtained by MRS with the anatomical and pathological information obtained from MRI. MRS has been used as a method for assessing both neuronal viability and demyelination. The use of MRS in neurologic disease has grown rapidly over the past decade, but the use of MRS in the setting of environmental insult to the brain is quite new. Earlier MRS studies of lead-exposed individuals do suggest effects on brain metabolites, but these studies focused on small numbers of very highly exposed children (Meng et al. 2005; Trope et al. 1998, 2001). Examination of the effects of exposure levels to which a much larger population would be exposed have not been done. We assessed the association between cumulative exposure to lead—as measured with K-X-ray fluorescence (KXRF) of bone lead—and brain metabolite ratios measured with MRS among participants in the Department of Veterans Affairs’ (VA) Normative Aging Study (NAS), a community-based cohort of elderly U.S. men with lead exposure similar to a similarly aged cross-section of the general U.S. population.

## Methods

### Study population

This research was conducted on a subgroup of the VA NAS, a multi-disciplinary longitudinal study of aging in men established by the VA in 1963 (Bell et al. 1966). The research herein was approved by the Human Subjects Committees of the Boston VA Medical Center, the Brigham and Women’s Hospital, and the Harvard School of Public Health. This cohort has been described in detail elsewhere (Hu et al. 1996). Briefly, healthy men from the general population in the greater Boston, Massachusetts, area were recruited in the 1960s. These men reported for medical examinations every 3–5 years, at which time they underwent clinical examinations and completed extensive health and lifestyle related questionnaires. The attrition rate for all causes has been < 1% annually and the response rate to mailed questionnaires that supplement onsite examinations has been > 80%.

Beginning in 1991 those who gave their informed consent presented to the Ambulatory Clinical Research Center of the Brigham and Women’s Hospital for a KXRF measurement of lead content in the tibia and patella bones. We selected 105 men from the lowest quintile of patella bone lead and 119 from the highest quintile to participate in the MRS study. Of these, 13 from the lowest and 12 from the highest quintiles were either too sick or ineligible for MRS because of possible metal in their body or we were unable to contact them (*n* = 27 from the lowest and 41 from the highest quintile). Of the remaining 65 men from the lowest and 66 from the highest quintile, 15 (23.1%) and 16 (24.2%), respectively, agreed to undergo an MRS scan. These scans were performed from 2002 through 2004. Bone lead for these 31 participants was measured between 1994 and 1999.

### Bone lead levels measured by KXRF

Bone lead was measured at two anatomical sites—the midtibial shaft and the patella—with an ABIOMED KXRF instrument (ABIOMED, Danvers, MA) as described previously (Hu et al. 1998). The tibia and patella have been targeted for bone lead research because these two bones are primarily cortical and trabecular bone, respectively, with different ramifications in terms of toxicity (Hu et al. 1998). A 30-min measurement was taken at the midshaft of the left tibia and at the left patella, after each region had been washed with a 50% solution of isopropyl alcohol. The tibial midshaft was taken as the midpoint between the tibial plateau and the medial malleolus. The KXRF beam collimator was sited perpendicular to the flat bony surface of the tibia and at 30° in the lateral direction for the patella.

### Magnetic resonance spectroscopy

MRI and ^1^H-MRS were performed on a 3T scanner (Signa LX; General Electric, Waukesha, WI). Anatomical MR images were obtained in the coronal plane using a 3D magnetization–prepared rapid gradient echo (MPRAGE) sequence developed in-house. The sequence was acquired with a field of view of 24 cm, 32 slices with a 3-mm slice thickness, 256 × 256 matrix size 600-msec inversion time, repetition time (TR) of 8 msec, echo time (TE) of 3 msec, a bandwidth of 32 kHz, and 1 excitation. These images were used to select graphically the left and right hippocampus for solvent suppressed ^1^H-MRS. Single-voxel ^1^H-MRS of the hippocampus was performed using point-resolved spectroscopy (PRESS). The ^1^H spectra (2 × 2 × 2 cm^3^) were acquired with a repetition time of 2 sec, time to echo of 35 msec, spectral width of 5,000 Hz, 2,048 time points, and 128 averages (4.3 min) and an eight-step phase cycling scheme. Crusher gradients of 32 mT/m amplitude (80% of the full-scale system gradient amplitude) and a duration of 4 msec (maximum crusher width) were equally spaced around the 180° pulses with 10-msec spacing. Spatial saturation pulses were applied at the edge of the PRESS voxel to minimize contamination of signal from outside the prescribed voxel. Linear shims were used to correct the B0 inhomogeneity across the investigated voxel. Spectral analysis was performed using LC-Model (Stephen Provencher Inc., Oakville, Ontario, CN) embedded in the Spectroscopy Analysis by GE package (SAGE) (GE Medical Systems, Milwaukee, WI). After any upgrade to the scanner we validated MRS results on phantoms. The output of LC-Model gave both the metabolite ratios (expressed as relative concentration taking into account the number of protons in each compound) and their standard deviations. Peak assignments for the different metabolites were those routinely used (Kreis et al. 1997).

### Data analysis

Analysis was performed on the main metabolites detected by MRS, that is, *N*-acetylaspartate (NAA), myoinositol (mI), and choline (Cho), all expressed as a ratio with creatine (Cr), which is generally stable in the brain and normalizes the relative intensities. When modeling the overall association between bone lead biomarkers and metabolite ratios, we included measurements of metabolite ratios from the hippocampus on both sides of the brain of each participant in our analyses by using repeated measures, with a compound symmetry covariance structure, to account for the within subject correlation. The MRS scans were performed an average of 6.0 (SD = 1.2) years after the KXRF measurements; therefore, we report on additional analyses after adjusting each subject’s patella bone lead levels for the years between the bone measurement and the MRS scan based on a first-order exponential decay function with a half-life of 8 years as determined previously among NAS participants (Kim et al. 1997). We did not adjust tibia concentrations because the same previous study found that they did not change over time periods similar to those described here. Other data on subjects were taken from their most recent regular NAS visit. Interquartile ranges (IQR) are the values of a given distribution at the 25th and 75th percentiles.

## Results

**Figure 1:**
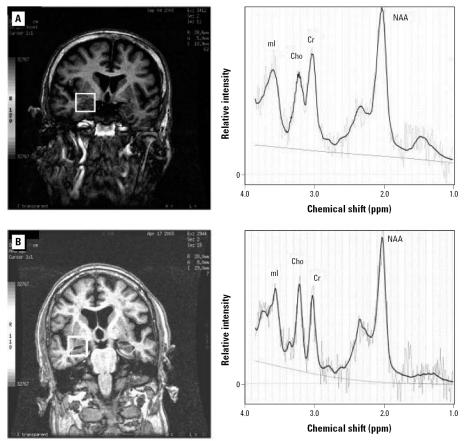
Example MRI and magnetic resonance spectroscopy spectra from participants in the low (*A*) and high (*B*) bone lead groups. The left panels show the region of interest outlined by a thick white box overlying the right hippocampal region on the MRIs. The right panels show the accompanying spectra with peaks for mI, Cho, Cr, and NAA indicated.

Demographic and health characteristics of NAS men selected for participation in this study are shown in Table 1 according to whether they participated and whether they were in the low- or high-patella lead group. When segregated by patella lead levels, there was little difference between those men that did and did not participate. None of the men who participated had been diagnosed with Alzheimer disease. In the complete NAS cohort we have previously reported inverse associations between bone lead and cognitive function (Weisskopf et al. 2004b, 2007; Wright et al. 2003), and in the small subset that participated in this MRS study, the Mini-Mental State Examination (Folstein et al. 1975) scores were higher among the low-lead group (mean, 26.9; SD = 2.0) than among the high-lead group (mean, 26.3; SD = 1.8). A representative scan showing the voxel of interest in the left hippocampus along with the proton spectra is shown in Figure 1. Among NAS men in the low-lead group the mean ± SD mI/Cr, NAA/Cr, and Cho/Cr ratios were 0.89 ± 0.29, 1.26 ± 0.38, and 0.33 ± 0.09, respectively. Among men in the high-lead group, the mean ± SD ratios were 0.99 ± 0.25, 1.24 ± 0.31), and 0.41 ± 0.29).

In univariate analyses, both patella and tibia bone lead were associated with a perturbation of the mI/Cr ratio (Table 2). Among men in the NAS with bone lead—from whom the 31 in this study were selected—the interquartile range of patella and tibia lead concentration is approximately 20 and 15 μg/g bone, respectively (Weisskopf et al. 2007). A 20-μg/g bone higher patella lead concentration and 15-μg/g bone higher tibia lead concentration were associated with an increase in mI/Cr ratio of 0.04 [95% confidence interval (CI), 0.00–0.08; *p* = 0.03] and 0.04 (95% CI, 0.00–0.08; *p* = 0.06), respectively. Each of these effects represents approximately 14% of the SD of the distribution of mI/Cr in the hippocampus among this group. mI is an important component of the phosphatidylinositol second messenger system, and lithium treatment has been reported to influence the brain levels of this metabolite (Silverstone et al. 2005). None of the subjects in our study used lithium.

**Figure 2:**
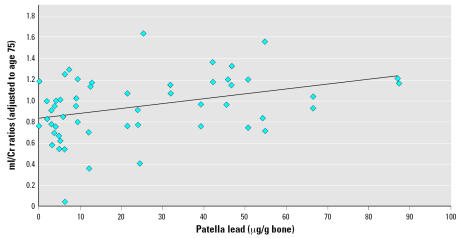
mI/Cr ratio (adjusted to 75 years of age) in the hippocampus by patella bone lead level (solid line is linear trend). Patella bone lead levels are adjusted to account for decay between time of bone lead measurement and MRS scan. mI/Cr ratios from the hippocampus on each side of the brain of participants are shown. *p* for trend = 0.04.

When we adjusted for age in the models, the effect sizes for both patella (0.05 per 20 μg/g) and tibia (0.05 per 15 μg/g) became stronger than in the crude analyses, but the precision was slightly reduced because of the high correlation between age and bone lead. None of the other metabolite ratios showed significant associations with either patella or tibia lead (Table 2). In analyses adjusted for age and using patella lead levels adjusted to account for decline in patella lead levels over the time between bone lead measurement and MRS, the results were also stronger than those in the main analyses: the effect estimate for a 20-μg/g bone patella lead increase was almost double (0.09; 95% CI, 0.00–0.18; *p* = 0.04) that in the age-adjusted main analysis. Figure 2 shows the individual mI/Cr ratios adjusted to 75 years of age indicating this association with patella lead.

Renal insufficiency can affect lead burden and possibly mI levels. Therefore, we also ran analyses after excluding the two men with serum creatinine > 1.5 mg/dL (one of whom was the participant who reported both a previous myocardial infarction and stroke) and a third man with diabetes, all three of whom were in the high-lead group. In these analyses the age-adjusted association between patella lead adjusted to account for decline in lead over time, and mI/Cr was even stronger than in the main analyses (effect estimate per 20 μg/g bone: 0.11; 95% CI, 0.02–0.19; *p* = 0.02). In addition, we ran analyses adjusting for hypertension (or use of hypertension medication) because these were reported more frequently in the high-lead group. In these analyses the age-adjusted association between patella lead adjusted to account for decline in lead over time and mI/Cr was also stronger than the main analyses (effect estimate per 20 μg/g bone: 0.13; 95% CI, 0.05–0.21; *p* = 0.002). Associations with other metabolite ratios remained nonsignificant in all of these additional analyses.

## Discussion

In this analysis of elderly men with lifetime exposures to lead similar to those of the general U.S. population of similar age, we found an association between an indicator of higher cumulative lead exposure—measured by lead concentration in bone—and increased mI/Cr ratios in the hippocampal region. There were no significant associations with any of the other metabolite ratios. Results were more pronounced when analyses were adjusted for age, although less precise because of high correlation between age and bone lead. However, when adjusting for age in analyses of patella lead adjusted for the time between the KXRF and MRS, the association with mI/Cr was both stronger and more precise. The magnitude of the increase in mI/Cr for an interquartile range higher bone lead concentration was about 14% of the SD of mI/Cr in this group.

This study is, to our knowledge, the first to examine the association of cumulative exposure to lead in a group of adults and metabolic changes in brain as measured with MRS. It is important to note that the exposure to lead in our subjects is not occupational but rather from the general environment and thus at levels that a much larger population of elderly adults worldwide would be expected to have. The few previous studies of lead exposure and MRS have focused on children. A study in China of 6 children with blood lead > 27 μg/dL and 6 children with blood lead < 10 μg/dL found lower NAA/Cr ratios in both the frontal cortex and hippocampus associated with the higher lead exposure (Meng et al. 2005). mI levels were not determined. A study in the United States of 16 children with blood lead levels between 23 and 65 μg/dL and 5 children with blood lead < 10 μg/dL also found reduced NAA/Cr, but no change in mI/Cr, in the frontal cortex associated with the higher lead exposure (Trope et al. 2001), which is similar to their previous report of two cousins with disparate lead exposure (Trope et al. 1998). In all these studies the exposures were high. The average blood lead concentration of children 12–19 years of age in the United States was 5.6 μg/dL in 2001–2002 (National Center for Environmental Health 2005). Differences from our current findings could be related to the age or exposure level differences from these previous studies. The only study in adults examined monozygotic twin brothers with markedly different lead levels—both blood and bone—although they were dramatically elevated in both twins as a result of occupational exposure (Weisskopf et al. 2004a). The NAA/Cr ratio in the hippocampus was found to be lower in the twin with the higher lead levels. mI levels were not determined. A principal difference in the present study is that the exposures were chronic and low level. Thus, the fact that changes in mI/Cr but not in NAA/Cr were detected could suggest that mI/Cr is a more sensitive indicator than other metabolite ratios of chronic low-level lead exposure in adults.

In the cortex, NAA is located in neuronal cell bodies, and a decrease in NAA is generally accepted as an indicator of neuronal damage and loss (Ross et al. 2006). In contrast, mI is found primarily in glial cells. Inositol polyphosphates are important neuronal signaling molecules, but their concentrations in the brain relative to mI itself are quite small and they do not contribute to the MRS peak assigned to mI, which is composed mostly of mI alone and mI–monophosphate (Ross 1991). Although the pathological significance of elevated mI is not completely clear, it has been proposed to reflect increased numbers of glial cells (Brand et al. 1993; Ross et al. 2006). Elevated mI and decreased NAA have been widely reported in patients with Alzheimer disease compared with normal controls in neocortical, limbic cortical, and hippocampal regions (Ross et al. 2006). Although other dementias share the feature of a decrease in NAA/Cr, the increase in mI/Cr is a relatively distinctive aspect of Alzheimer disease and has been suggested as indicative of gliosis (Kantarci et al. 2004; Ross et al. 2006). It has been proposed that the initial MRS changes in the pathologic progression of Alzheimer disease is an increase in mI/Cr and that a decrease in NAA/Cr develops later in the disease course, based on data from the posterior cingulate gyrus (Kantarci et al. 2000). This is supported by data indicating that glial proliferation and plaque formation occur in preclinical Alzheimer disease (Mackenzie et al. 1995; Morris and Price 2001), whereas neuronal loss may not occur at this stage (West et al. 2004). The difference in mI/Cr reported between Alzheimer disease patients and normal controls (Kantarci et al. 2004; Ross et al. 2006) is similar in magnitude to the change we found associated with an interquartile range higher patella bone lead concentration.

Little is known about the structural and functional correlates of lead-related brain dysfunction in humans. Largely from *in vitro* and animal work it is known that lead accumulation in the brain after exposure is preferentially in glia rather than in neurons (Lindahl et al. 1999; Tiffany-Castiglioni et al. 1989), and chronic lead exposure in rats has been found to induce astrogliosis in the hippocampus and cerebellum (Selvin-Testa et al. 1994). The astroglial response to lead exposure may in fact be an initial attempt to protect neurons from injury (Struzynska et al. 2005). Our results are consistent with these animal studies. Although lead most certainly adversely affects neurons as well, it may be that, compared with effects on glial cells, higher levels of lead exposure are needed before those effects can be detected as a reduced NAA/Cr ratio in humans with MRS, and, therefore, the glial effects may be an earlier MRS marker of effects of chronic lead exposure.

A limitation to this study is the small sample size for study of the effects of relatively low levels of lead exposure. Nonetheless, this is the largest such study to date. Further study of a larger cohort would provide better power to detect possible other associations. Although the participation rate was low, those that participated and those that did not appeared similar in many respects. An additional concern is that participants in the high-lead group were somewhat older than those in the low-lead group. However, the mI/Cr ratio appears to decrease with normal aging (Ross et al. 2006); thus our finding is in the opposite direction of the possible age bias. Indeed, our results were stronger when adjusted for age. Another concern is that renal insufficiency can affect lead burden and may influence mI levels. However, our results were stronger when the two men with serum creatinine > 1.5 mg/dL and one man with diabetes were excluded from analyses. Last, the region of interest analyzed, while centering on the hippocampus, includes some surrounding tissue. Therefore, to the extent that the effects of lead on metabolite ratios differ within and immediately outside the hippocampus, observed associations would be weakened.

In summary, we find an association between increased cumulative lead exposure and increased mI/Cr ratios in the hippocampus among a group with general population levels of lead exposure. We did not see significant associations between lead and any of the other metabolite ratios. These data suggest that in humans, as has been seen in animal studies, glial effects may also be the first or most sensitive indicators of adverse effects of cumulative lead exposure in adults, and these changes are similar to what is seen in early stages of Alzheimer disease.

## Figures and Tables

**Table 1:** Characteristics by participation status and patella lead level.

	Did not participate Patella lead level		Participated Patella lead level	
Characteristic	Low (*n* = 90)	High (*n* = 103)	Low (*n* = 15)	High (*n* = 16)
Mean age in years ± SD at MRS^a^	73.0 ± 6.2	78.0 ± 5.9	73.2 ± 4.7	80.7 ± 6.1
Mean years ± SD of education	15.3 ± 2.5	13.2 ± 2.6	16.3 ± 3.1	13.4 ± 2.6
Mean ± SD serum creatinine (mg/dL)	1.1 ± 0.5	1.1 ± 0.3	1.0 ± 0.2	1.2 ± 0.4
Mean ± SD systolic blood pressure (mmHg)	134 ± 16	134 ± 17	133 ± 15	135 ± 20
Diabetes, *n* (%)	7 (8)	5 (5)	0	1 (6)
Hypertension, *n* (%)	28 (31)	36 (35)	4 (27)	7 (44)
Taking hypertension medication, *n* (%)	9 (10)	15 (15)	1 (7)	6 (38)
Myocardial infarction, *n* (%)	0	6 (6)	0	1 (6)
Stroke, *n* (%)	0	3 (3)	0	1 (6)
Median (IQR) patella lead (μg/g bone)	10 (6–14)	48 (44–60)	9 (5–15)	63 (43–86)
Median (IQR) tibia lead (μg/g bone)	11 (7–17)	32 (24–41)	13 (9–17)	41 (38–59)

Hg, mercury.

a Age on 1 January 2002 for those without MRS.

**Table 2:** Crude effect estimates^a^ for tibia and patella lead on MRS metabolite ratios in the hippocampus.

Metabolite	Effect estimate	95% CI	*p*-Value
Patella lead			
mI/Cr	0.04	0.00 to 0.08	0.03
NAA/Cr	0.00	−0.05 to 0.05	0.99
Cho/Cr	−0.01	−0.04 to 0.02	0.42
Tibia lead			
mI/Cr	0.04	0.00 to 0.08	0.06
NAA/Cr	0.00	−0.05 to 0.06	0.90
Cho/Cr	−0.01	−0.04 to 0.03	0.66

a Effect estimates were per 20 and 15 μg/g increases in patella and tibia lead concentration, respectively, which are the interquartile ranges for those measures in the parent Normative Aging Study population from which the subjects in this study were selected. Because of missing metabolite values the number of subjects in analyses of mI/Cr is 29.
